# Synergistic effects of *APOE* and sex on the gut microbiome of young EFAD transgenic mice

**DOI:** 10.1186/s13024-019-0352-2

**Published:** 2019-12-20

**Authors:** Juan Maldonado Weng, Ishita Parikh, Ankur Naqib, Jason York, Stefan J. Green, Steven Estus, Mary Jo LaDu

**Affiliations:** 1 Department of Anatomy and Cell Biology, College of Medicine, University of Illinois at Chicago, Chicago, IL 60612 USA; 2 Department of Physiology and Sanders-Brown Center on Aging, University of Kentucky, Lexington, KY USA; 3 Research Resources Center, University of Illinois at Chicago, Chicago, IL USA

**Keywords:** Alzheimer’s disease, Gut microbiome, *APOE* genotype, Sex

## Abstract

**Background:**

Alzheimer’s disease (AD) is a fatal neurodegenerative disease. *APOE4* is the greatest genetic risk factor for AD, increasing risk up to 15-fold compared to the common *APOE3.* Importantly, female (♀) *APOE4* carriers have a greater risk for developing AD and an increased rate of cognitive decline compared to male (**♂**) *APOE4* carriers. While recent evidence demonstrates that AD, *APOE* genotype, and sex affect the gut microbiome (GM), how *APOE* genotype and sex interact to affect the GM in AD remains unknown.

**Methods:**

This study analyzes the GM of 4-month (4 M) ♂ and ♀ E3FAD and E4FAD mice, transgenic mice that overproduce amyloid-β 42 (Aβ42) and express human *APOE3*^*+/+*^ or *APOE4*^*+/+*^. Fecal microbiotas were analyzed using high-throughput sequencing of 16S ribosomal RNA gene amplicons and clustered into operational taxonomic units (OTU). Microbial diversity of the EFAD GM was compared across *APOE,* sex and stratified by *APOE +* sex, resulting in 4-cohorts (♂E3FAD, ♀E3FAD, ♂E4FAD and ♀E4FAD). Permutational multivariate analysis of variance (PERMANOVA) evaluated differences in bacterial communities between cohorts and the effects of *APOE +* sex. Mann-Whitney tests and machine-learning algorithms identified differentially abundant taxa associated with *APOE* + sex.

**Results:**

Significant differences in the EFAD GM were associated with *APOE* genotype and sex. Stratification by *APOE* + sex revealed that *APOE*-associated differences were exhibited in ♂EFAD and ♀EFAD mice, and sex-associated differences were exhibited in E3FAD and E4FAD mice. Specifically, the relative abundance of bacteria from the genera *Prevotella* and *Ruminococcus* was significantly higher in ♀E4FAD compared to ♀E3FAD, while the relative abundance of *Sutterella* was significantly higher in ♂E4FAD compared to ♂E3FAD. Based on 29 OTUs identified by the machine-learning algorithms, heatmap analysis revealed significant clustering of ♀E4FAD separate from other cohorts.

**Conclusions:**

The results demonstrate that the 4 M EFAD GM is modulated by *APOE* + sex. Importantly, the effect of *APOE4* on the EFAD GM is modulated by sex, a pattern similar to the greater AD pathology associated with ♀E4FAD. While this study demonstrates the importance of interactive effects of *APOE* + sex on the GM in young AD transgenic mice, changes associated with the development of pathology remain to be defined.

## Background

The gut microbiome (GM), the collective genome of gastrointestinal bacteria, is an integral component of human physiology [1–5]. Recent studies link dysbiotic GM profiles with neurological disorders, with multiple sclerosis the first identified [6–12]. While subsequent studies have linked dysbiosis with Alzheimer’s disease (AD) pathology [13–22], the effects of AD risk factors, specifically *APOE* genotype, sex and their interaction, on the GM remain unclear.

The *APOE4* genotype is the greatest genetic risk factor for AD, increasing risk up to 15-fold compared to the more common *APOE3* genotype [23, 24]. Apolipoprotein E (apoE) is a member of the apolipoprotein family, the protein components of lipoproteins. Both humans and AD transgenic (−Tg) mice with *APOE4* exhibit an increase in amyloid-β (Aβ) peptide accumulation, both as amyloid plaques, a hallmark of the disease, and small soluble aggregates. Thus, one explanation for the *APOE4*-associated AD risk is a loss of function in Aβ clearance. Tran and colleagues demonstrated significant differences between the GM of human *APOE3* and *APOE4* carriers, as well as differences between the GM of *APOE3* and *APOE4* targeted replacement (−TR) mice [25]. These differences were attributed to a loss of apoE4 function in lipid homeostasis*,* as *APOE4* is associated with higher levels of cholesterol, triglycerides and low-density lipoproteins compared to *APOE3* [26–29], changes that significantly affect the GM [30–37]. Sex is another risk factor for AD as females (♀) exhibit almost two-fold greater lifetime AD risk compared to males (**♂**) [38]. Additionally, sex plays an important role in the GM as the bacterial composition and metabolic function differ significantly between **♂** and ♀ [37, 39–46]. Importantly, ♀*APOE4* carriers have a greater lifetime risk for developing AD, an increased rate of cognitive decline and an accelerated accumulation of Aβ compared to **♂***APOE4* carriers [47–61]. While the underlying mechanism is unclear, evidence suggests this interaction modulates the GM.

EFAD-Tg mice [62] overexpress Aβ42 via five familial AD (FAD) mutations [63] and express h-*APOE3* or *APOE4*, allowing for the study of the interaction among AD risk factors [64–66]. EFAD mice expressing the *APOE4*^+/+^ genotype (E4FAD), compared to E3FAD mice, exhibit increased behavioral deficits, Aβ deposition and neuroinflammation. Importantly, these differences are reproduced in ♀ vs **♂**EFAD mice, resulting in 4 pathologically-distinct cohorts when the EFAD mice are stratified by *APOE* + sex (♀E4FAD > ♂E4FAD = ♀E3FAD > ♂E3FAD), a phenotype that develops with age [65, 66]. For this study, we focused on 4 M EFAD mice to evaluate the interactive effects of *APOE* + sex on the GM at an age prior to, or early in, the development of pathology. Microbial analysis of fecal samples demonstrated that *APOE* + sex have a significant effect on the GM at various taxonomic levels.

## Methods

### Mouse model

As previously described, the EFAD (5xFAD^+/−^/*APOE*^+/+^) mice are homozygous for *APOE2*, *APOE3*, or *APOE4* and heterozygous for the 5x familial AD (5xFAD) mutations [62, 63]. Although *APOE2* is considered neuroprotective, 100% of *APOE2*^+/+^ mice have type III hyperlipoproteinemia, compared to only 15% of human ε2/2 carriers [67–69]; thus, E2FAD mice were excluded from the current study. At 4 M, fecal samples were obtained from the 4 cohorts (9 ♂E3FAD, 8 ♂E4FAD, 19 ♀E3FAD, 12 ♀E4FAD) by individually placing mice in clean disposable Styrofoam cups. Feces were flash frozen and stored at − 80 °C until DNA isolation.

### Bacteria identification

Fecal DNA was isolated using a PowerSoil DNA isolation kit (Mo Bio Laboratories) and DNA concentrations determined by UV absorbance (Nanodrop, ThermoFisher). The V4 variable region of 16S ribosomal RNA gene was PCR-amplified using target-specific primers containing bar codes and linker sequences [70]. PCR reaction conditions included an initial denaturation step of 30 s (s) at 98 °C, followed by 28 cycles of 10s at 98 °C, 15 s at 60 °C, 30s at 72 °C, and a final elongation step of 7 min at 72 °C. The PCR master mix (20 μl volume) contained 100 ng of DNA template, 0.5 μM forward and reverse primers, Phusion Hot Start DNA polymerase and high-fidelity buffer (New England Biolabs), dNTPs and sterile water. Results were checked by polyacrylamide gel electrophoresis and samples pooled in equimolar ratio. The samples were sequenced on an Illumina MiSeq sequencer at the University of Kentucky Advanced Genetic Technologies Center, with sequence merging, trimming, chimera removal, clustering and annotation performed using the software package QIIME [71]. The Greengenes database was implemented for Operational Taxonomic Unit (OTU) annotation at a threshold of 97% sequence similarity [72]. To avoid effects of uneven sequencing depth [73], datasets were rarified to 3000 sequences/sample prior to analysis. For statistical analyses, OTUs with a frequency below 0.1% across the dataset were removed [71].

### Data analysis

The Shannon H α-diversity index was used to assess bacterial richness and evenness. The interaction between *APOE* + sex in α-diversity measures was evaluated using a mixed effects model, similar to a two-way analysis of variance (ANOVA), that analyzes repeated measures with missing values. This analysis was performed in the software package GraphPad Prism (version 8.2.0). For β-diversity, permutational ANOVA (PERMANOVA) was used to compare microbial community structure within and among the EFAD cohorts based on Bray-Curtis dissimilarity [74, 75]. Pair-wise PERMANOVA was used to assess the effect of the interaction among universal biological variables on the microbiome composition [76]. Principal coordinate analysis plots (PCoA; Bray-Curtis distances) with 95% confidence ellipses were used to visualize microbial communities [75, 77, 78]. The Mann-Whitney U (MWU) test under the Monte Carlo simulation, corrected with Benjamini-Hochberg False Discovery Rate (*p* < 0.05), was used to identify differentially abundant taxa associated with *APOE* + sex at the taxonomic level of genus. The Random Forest based Boruta algorithm was used to determine OTUs significant in distinguishing samples by *APOE* + sex compared to randomly generated probes or “shadow scores” [79]. Heatmaps were generated using the R package, “pheatmaps”, calculating the Euclidean distance among cohorts.

## Results and discussion

Mouse fecal microbial community structure was analyzed using high-throughput sequencing of 16S rRNA gene amplicons, followed by sequence clustering (97% similarity) into a total of 2063 OTUs. No significant difference in α-diversity (Shannon H index) was observed between E3FAD and E4FAD mice (*p* = 0.975; Additional file 1: Figure S1A) or between ♂EFAD and ♀EFAD (*p* = 0.949; Additional file 1: Figure S1B). In comparing across cohorts stratified by *APOE* + sex, Shannon H indices were significantly higher in ♂E4FAD and ♀E3FAD, compared to ♂E3FAD and ♀E4FAD (*p* < 0.05; Additional file 1: Figure S1C). Additionally, the interaction of *APOE* + sex significantly modulated α-diversity measures (*p* < 0.05; Additional file 1: Figure S1C), suggesting that analyses by *APOE* genotype or sex alone will mask effects on microbial community structure.

**Fig. 1:**
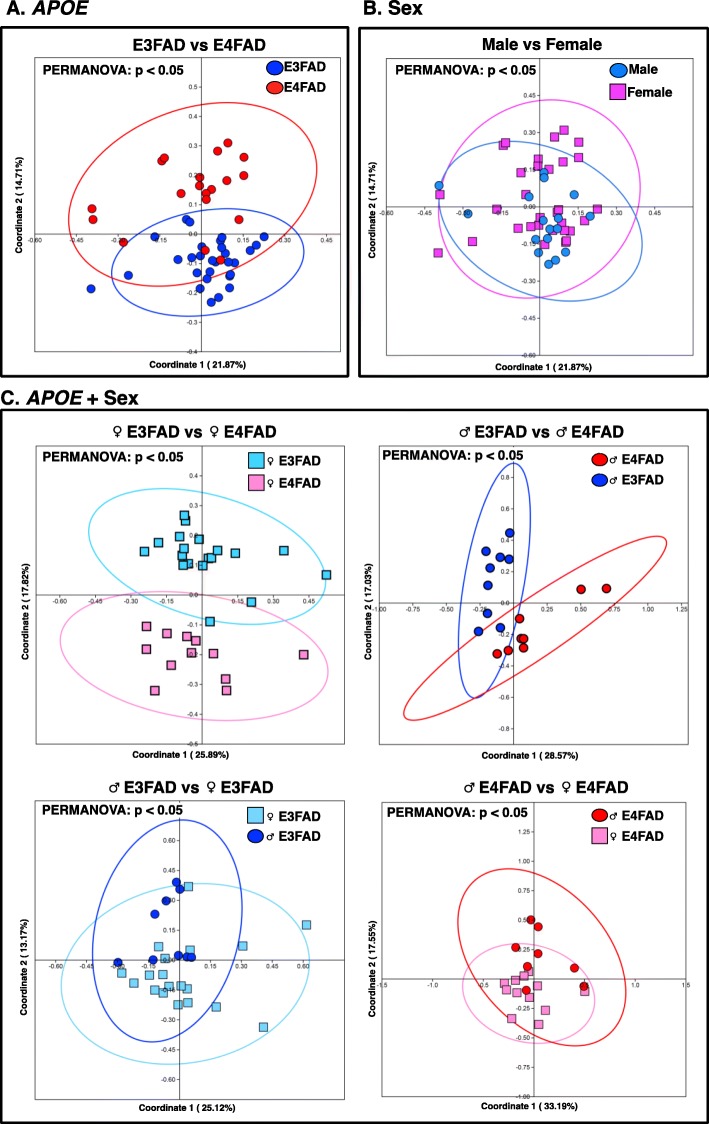
Differences in microbial community between EFAD mice stratified by *APOE*, sex and *APOE* + sex. Analysis of β-diversity associated with (**a**) *APOE*, (**b**) sex and (**c**) *APOE* + sex in the GM of 4 M EFAD mice. PCoA plots with 95% confidence ellipses were generated based on the Bray-Curtis dissimilarity. Significant differences between cohorts were determined by PERMANOVA, with significance (**bold**) defined by *p* < 0.05. Additional file 1: Table S1 contains the complete PERMANOVA dataset

Differences in microbial community structure between EFAD cohorts (β-diversity) were examined with PERMANOVA (Additional file 3: Table S1) and visualized with PCoA plots (Fig. 1). At the taxonomic level of OTU, significant differences in microbial communities were observed between E3FAD and E4FAD mice (*p* < 0.05; Fig. 1a) and between ♂EFAD and ♀EFAD mice (*p* < 0.05; Fig. 1b). Differences associated with *APOE* genotype were also exhibited in the taxonomic levels of Family and Genus (Additional file 3: Table S1A), suggesting that *APOE* genotype is an important modulator of the GM, consistent with findings in *APOE*-TR mice [25]. Importantly, the interaction between *APOE* + sex significantly modulated the GM across taxonomic levels of Family, Genus and OTU (*p* < 0.05; Additional file 3: Table S1A). Comparisons at the OTU level among samples stratified by *APOE* + sex demonstrated significant differences between ♂E4FAD and ♂E3FAD mice (*p* < 0.05; Fig. 1c), and between ♀E4FAD and ♀E3FAD mice (*p* < 0.05; Fig. 1c), indicating that the effect of *APOE* genotype is consistent across sex. Furthermore, significant differences associated with sex were observed between ♂E4FAD and ♀E4FAD and between ♂E3FAD and ♀E3FAD (*p* < 0.05; Fig. 1c). These data demonstrate that the *APOE* genotype interacts with sex, leading to sex differentiation in E3FAD and E4FAD mice. While a recent paper by Dodiya and colleagues demonstrated no sex effect on α- or β-diversity in FAD-Tg mice that express mouse *APOE* [80], the current findings may suggest that the sex effect is specific to carriers of human *APOE*. This mirrors the synergistic effects of ♀sex and *APOE4* genotype on AD risk in humans, greatest in ♀*APOE4* > **♂***APOE4* [47–50].

**Fig. 2:**
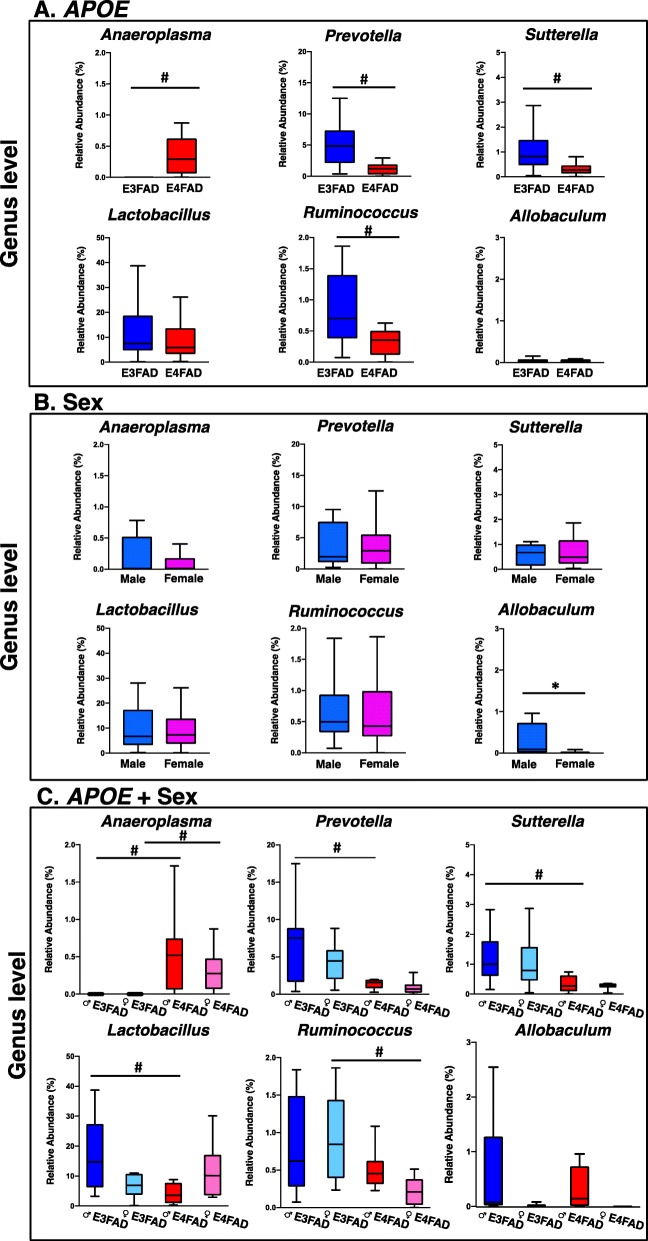
Relative abundance of bacterial genera in EFAD mice stratified by *APOE*, sex, *APOE* + sex. Significantly different relative abundance of genus-level bacterial taxa associated with (**a**) *APOE*, (**b**) sex and (**c**) *APOE* + sex, identified by Mann-Whitney U test with a Monte Carlo Simulation corrected for false discovery rate (*p < 0.05 vs sex; #*p* < 0.05 vs genotype). Tukey plots show the median and interquartile range, with outliers removed from the graph. Significantly different relative abundance of unclassified genera and taxa from other taxonomic levels are found in the Additional file 4: Table S2.

A taxon-by-taxon analysis at the genus level was performed to identify microbial genera significantly different between cohorts. The relative abundance of the genera *Prevotella*, *Ruminonoccous* and *Sutterella* were significantly higher in E3FAD mice compared to E4FAD mice, while the relative abundance of *Anaeroplasma* was significantly lower (Fig. 2a). Interestingly, FAD-Tg mice also exhibited significantly higher relative abundance of *Anaeroplasma* compared to wild-type mice [81, 82], suggesting that *Anaeroplasma* may have a role in AD pathology. Tran and colleagues demonstrated that *APOE4*-TR mice exhibit greater relative abundance of bacteria from the genera *Mucispirillum*, *Desulfovibrio*, *Butyricicoccus* and lower relative abundance of *Bacteroides*, *Alistipes*, *Johnsonella* compared to *APOE3*-TR mice [25]. Thus, our results together suggest that the effects of *APOE* genotype on the GM is modulated by AD pathology. Additionally, Org and colleagues determined that *Allobaculum*, *Anaeroplasma* and *Erwinia* are the most abundant genera in ♂mice relative to ♀mice [83]. Similarly, ♂EFAD exhibited a significantly greater relative abundance of *Allobaculum* compared to ♀EFAD (Fig. 2b). Comparing the stratified cohorts, the fecal microbiota of ♂E4FAD mice had lower relative abundance of *Sutterella* and *Lactobacillus* compared to ♂E3FAD. ♀E4FAD mice had lower relative abundance of *Prevotella* and *Ruminococcus* compared to ♀E3FAD (Fig. 2c). Similarly, these differences are significant at the OTU level (Additional file 4: Table S2). Therefore, the results suggest that the effect of *APOE* genotype on differentially abundant bacteria is modulated by sex, as specific genera and OTUs are significantly different in males or females.

Compared to ♀E3FAD mice, ♀E4FAD mice exhibited a lower relative abundance of bacterial genera associated with short chain fatty acid (SCFA) production, including *Prevotella* and *Ruminococcus* [84–89]. The GM is crucial for the production of SCFAs that, while the underlying mechanism is not completely understood, serve as energy sources for intestinal epithelial cells, regulators of plasma lipid levels, and modulators of immune cells [90–95]. The current results suggest a metabolic dysfunction in the ♀E4FAD GM. However, metabolomic and metagenomic analyses will be required to interpret accurately the interactive effects of *APOE* + sex on the metabolic function of the EFAD GM.

**Fig. 3:**
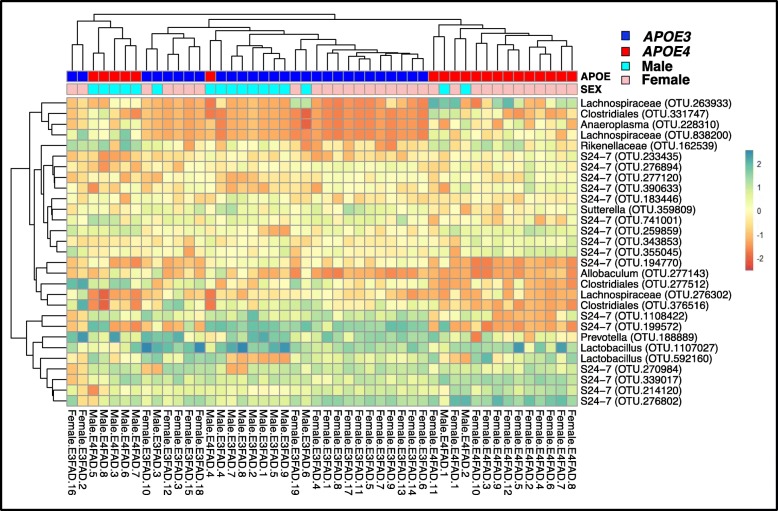
Two-way clustered heatmap of microbial OTUs from EFAD mice stratified by *APOE* + sex. Heatmap generated with hierarchical clustering (Euclidean distance, complete linkage) based on bacterial OTUs identified by Boruta, a Random Forest based machine learning algorithm (Additional file 2: Fig. S2)

The Boruta algorithm identified 29 OTUs significant in distinguishing EFAD samples by *APOE* + sex (Additional file 2: Figure S2). These 29 bacterial OTUs were annotated at varying taxonomic levels, including the genera *Prevotella*, *Lactobacillus*, *Allobaculum, Anaeroplasma*, and *Sutterella*, consistent with the results of differentially abundant bacteria (Fig. 2). Based on the abundance of these 29 OTUs, a hierarchical heatmap demonstrates that EFAD samples clustered by *APOE* + sex (Fig. 3). Clustering of ♀E4FAD samples is further demonstration that the murine GM is affected by a specific interaction between *APOE4* genotype and ♀sex, consistent with human ♀*APOE4* carriers exhibiting greater AD risk compared to ♂*APOE4* carriers [47–50].

## Conclusions

This short report demonstrates: 1) the EFAD GM is modulated by *APOE* + sex, 2) the synergistic effects of ♀sex and *APOE4* genotype yield a specific GM profile in ♀E4FAD mice, and 3) clustering samples by only *APOE* genotype or sex masks the interactive effects of *APOE* + sex on the EFAD GM. Notably, these findings are consistent with AD readouts from EFAD mice varying in severity of pathology by *APOE* + sex, including behavioral deficits, Aβ deposition and neuroinflammation greatest in ♀E4FAD > ♂E4FAD = ♀E3FAD > ♂E3FAD [65, 66]. Therefore, the GM would potentially serve as an AD readout, reflecting the interaction between *APOE* + sex. Although the use of 16S rRNA sequencing has more limited taxonomic resolution than shotgun metagenome sequencing [96], 16S rRNA sequencing is sufficiently robust to identify significant effects on the GM. This study demonstrates the importance of stratifying the EFAD population by *APOE* + sex to better understand the relationship between AD and the GM. Future studies will examine the composition and metabolic function of the GM throughout the development of EFAD pathology through the use of metagenomic and metabolomic analyses. In conclusion, the interactive effects of *APOE* + sex on AD play an important role in modulating the GM composition, and the current report is the first step in identifying and understanding these effects.

## Supplementary information

**Additional file 1: Figure S1.** Analysis of α-diversity of EFAD mice stratified by *APOE*, sex, *APOE* + sex. Based on bacterial evenness and richness, Shannon H index scores were generated and compared across EFAD mice stratified by (**A**) *APOE*, (**B**) sex and (**C**) *APOE* + sex with Mann-Whitney U test (*p < 0.05 vs sex; #*p* < 0.05 vs genotype). A mixed-model analysis was used to evaluate the interactive effects of *APOE* + sex on richness, evenness and α-diversity (¶ *p* < 0.05 vs *APOE* + sex).

**Additional file 2: Figure S2.** Boruta-identified bacterial OTUs from EFAD mice stratified by *APOE* + sex. Implementing the R package “randomForest”, Boruta is a feature-selection algorithm that determined the OTUs that were significant in distinguish samples by *APOE* + sex compared to randomly generated probes (“shadow scores” in blue). Significance is defined by a z-score > max shadow z-score (green; listed in the table). OTUs with a z-score that trends towards significance are labeled in yellow.

**Additional file 3: Table S1.** Permutational multivariate analysis of variance (PERMANOVA) of EFAD mice stratified by *APOE*, sex, *APOE* + sex. (A) PERMANOVA was used to assess the effect of the interaction between universal biological variables on the microbiome composition at various taxonomic levels. *P*-values were obtained using 9999 permutations under a reduced model. Pseudo-F ratio is defined by the difference between cohorts over the difference within each cohort and the degrees of freedom. Each term is contributing a fixed component to the overall model. Estimated sizes of components of variation are multivariate analogs to the classical ANOVA unbiased estimators. Significance (**bold**) is defined by a *p* < 0.05. (B) As the interaction between *APOE* + sex is significant, pair-wise PERMANOVAs at the OTU level evaluated the effects of *APOE* on β-diversity within ♂EFAD and ♀EFAD mice, and the effects of sex in E3FAD and E4FAD. Significance (**bold**) is defined by a *p* < 0.05.

**Additional file 4: Table S2.** Results of Mann-Whitney U tests at specific taxonomic levels in EFAD mice. Significantly different relative abundance of bacterial genera associated with *APOE*, sex, and *APOE* + sex, identified by Mann-Whitney U under the Monte Carlo Simulation corrected for false discovery rate (*p* < 0.05) at the levels of Phylum, Class, Order, Family, Genus and OTU.

## Data Availability

The datasets used and/or analyzed during the current study are available from the corresponding author on reasonable request. Raw sequence data files were submitted in the Sequence Read Archive (SRA) of the National Center for Biotechnology Information (NCBI). The BioProject identifier of the samples is PRJNA556445.

## Abbreviations

AD: Alzheimer’s disease
apoE: Apolipoprotein E
Aβ: Amyloid-β
FAD: Familial AD
GM: Gut microbiome
MWU: Mann-Whitney U
OTU: Operational taxonomic units
Perm: Permutation
PERMANOVA: Permutational multivariate analysis of variance
SCFA: Short chain fatty acid
Tg: Transgenic
