# Supplementary material for: Synergistic effects of APOE and sex on the gut microbiome of young EFAD transgenic mice
Source: Mol Neurodegener. 2019 Dec 20;14:47. doi: 10.1186/s13024-019-0352-2 (PMC6923910; doi:10.1186/s13024-019-0352-2)

A. *APOE*

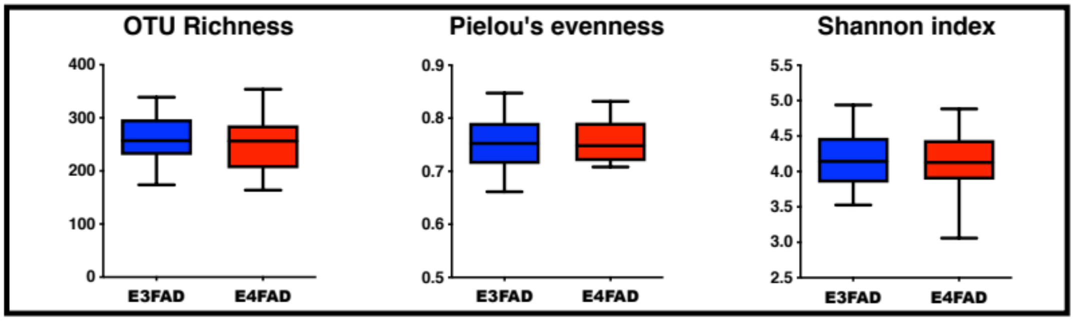

B. Sex

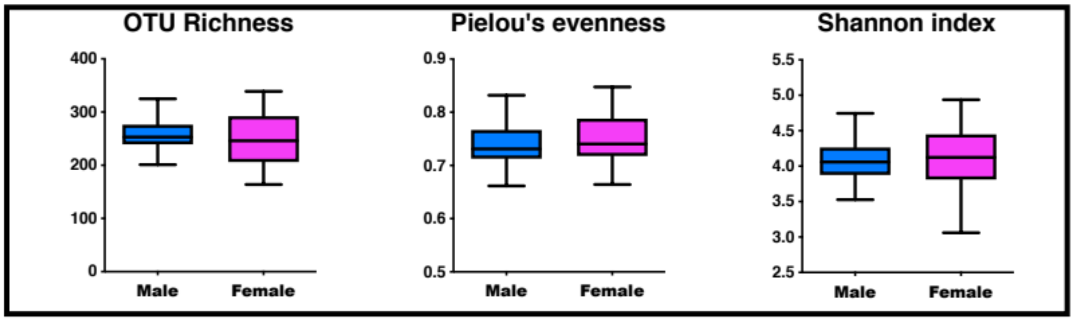

C. *APOE* + Sex

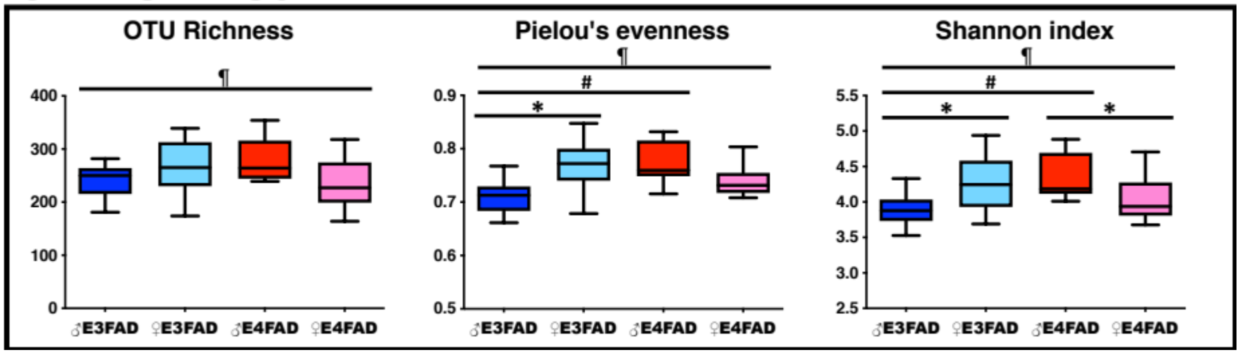

Supplement: Supplementary file 1 [file 13024_2019_352_MOESM1_ESM.docx]
