# Supplementary material for: Synergistic effects of APOE and sex on the gut microbiome of young EFAD transgenic mice
Source: Mol Neurodegener. 2019 Dec 20;14:47. doi: 10.1186/s13024-019-0352-2 (PMC6923910; doi:10.1186/s13024-019-0352-2)
Supplement: Supplementary file 2 [file 13024_2019_352_MOESM2_ESM.docx]

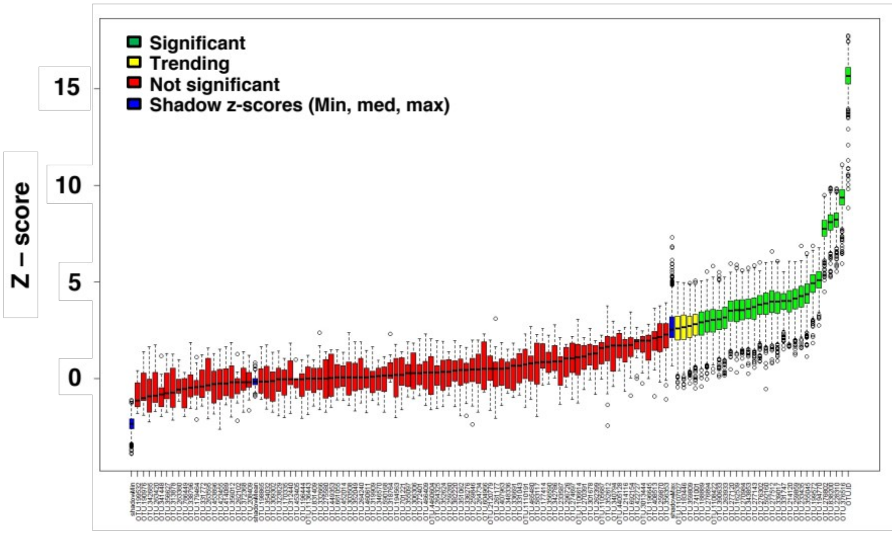

OTU.ID	Taxon
OTU.331747	o__Clostridiales
OTU.194770	f__S24-7
OTU.228310	g__Anaeroplasma
OTU.276302	f__Lachnospiraceae
OTU.277143	g__Allobaculum
OTU.233435	f__S24-7
OTU.277120	f__S24-7
OTU.838200	f__Lachnospiraceae
OTU.183446	f__S24-7
OTU.276894	f__S24-7
OTU.343853	f__S24-7
OTU.390633	f__S24-7
OTU.376516	o__Clostridiales
OTU.359809	g__Sutterella
OTU.355045	f__S24-7
OTU.263933	f__Lachnospiraceae
OTU.277512	o__Clostridiales
OTU.259859	f__S24-7
OTU.162539	f__Rikenellaceae
OTU.741001	f__S24-7
OTU.214120	f__S24-7
OTU.1108422	f__S24-7
OTU.270984	f__S24-7
OTU.339017	f__S24-7
OTU.592160	g__Lactobacillus
OTU.199572	f__S24-7
OTU.276802	f__S24-7
OTU.188889	g__Prevotella
OTU.1107027	g__Lactobacillus
